# COVID-19 in China: Power, Transparency and Governance in Public Health Crisis

**DOI:** 10.3390/healthcare8030288

**Published:** 2020-08-22

**Authors:** Jinrui Zhang, Ruilian Zhang

**Affiliations:** 1 School of Public Administration, Hohai University, Nanjing 211100, China; jr_zhang@hhu.edu.cn; 2 Sustainable Minerals Institute, University of Queensland, Brisbane 4072, Australia

**Keywords:** public health crisis, government collaboration, crisis management, COVID-19

## Abstract

Public health crises are the “touchstone” to test the ability of national public health crisis governance. The public health crisis in the new era presents new characteristics: systematic, cross-border and uncertainty. The governance dilemma of a public health crisis generally emphasizes the joint participation and communication of different subjects, which is suspected of overlapping and redundancy, and lacks the auxiliary support of major public health crisis events. It dispels the significance of government-level cooperation. The essence of the public health crisis governance system is the chain law of stimulus–response. In combination with COVID-19 development in China, we track down the main reasons for the temporary disruption and the government’s response to this major public health crisis. We mainly examine the tension between the centralization of power in China’s governance structure and the effectiveness of local governance, and the control of local governments in information disclosure. The response to a public health crisis and the optimization of a decision-making mechanism should build tension between the centralization of power and effectiveness of local governance. It is suggested that government should disclose and share information to the public timely and pay more attention to the core value of order in crisis management.

## 1. Introduction

The world is facing a great change that has not happened in a century. The lack of global governance resources calls for diversified national governance practices and experience sharing [1,2]. National governance systems and governance capacity are not only reflected in the regular governance activities, but also reflected in the response level when encountering a major public health crisis [3,4]. A major public health crisis is the “touchstone” to verify the national governance system and governance capacity. From normal governance to response to abnormal public health crisis events, the governance system and governance capacity have undergone process evolution in time series, especially in major public health crisis events [5,6]. As a threatening situation, public health crises do not occur frequently, especially major public health crises. However, due to the sudden, uncertain and various erosion of the social body, public health crises have become normal in, or one of the essential contents of, human survival [7]. It is believed that public health crises are a serious threat to the basic value and behavior framework of social systems, and are a state in which critical decisions must be made under the dual conditions of timeliness and uncertainty [8]. It can be seen that in the new era, the research on the response to public health crises and the optimization of decision-making mechanisms are practical problems worthy of attention, which play an important and positive role in further preventing and resolving social risks [9,10].

In 2003, China encountered the first public health crisis in the new century, namely severe acute respiratory syndrome (SARS). After that, the study of public health crisis has seen great extension, involving multiple subjects and various contents. Experience told us that local government information disclosure in public health crisis events is critically important [11]. Further, accountability and inertia are the main incentive sources of local government crisis information disclosure, and the institutional logic of local government concealing crisis information lies in the macro-governance structure of upper and lower governance [12].

The research topics of public health crisis are diverse, and the research methods present a mixture of quantitative research and qualitative research in public management [13]. Among them, there are few literatures on intergovernmental cooperation, and most of them focus on the theme of social governance. Logically, they tend to tie the government cooperation as a whole and regard it as a constituent element of cooperative governance, lacking deep-seated exploration. In the public health crisis, government-level cooperation is very important, and especially in the face of a major public health crisis, government-level cooperation often determines the scope of a public health crisis, the frequency of occurrence and the governance effect. It can be said that a major public health crisis is a “big test” for government-level cooperation. Coronavirus disease 2019 (COVID-19) is showing new features. Major public health crises, such as COVID-19 spreading at the moment, are already spreading worldwide. Intergovernmental collaboration is still on the way, and the possible repression is related to political, economic and cultural factors. Reviewing the COVID-19 prevention and control and government-level collaboration process in China is conducive to the optimization of public health crisis response and decision-making mechanisms.

## 2. Power

### 2.1. New Features of Public Health Crisis

Public health events refer to the sudden occurrence of major infectious diseases, mass diseases of unknown origin, major food and occupational poisoning that cause serious damage to the physical and mental health of the public, as well as events that seriously affect the physical and mental health of the public caused by natural disasters, accident disasters or social security events [14]. In post-industrial society, under the sweeping of network elements, the social structure gradually tends to be networked. Internet technology not only promotes the convenience of information interaction and exchange, but also takes it as the thinking scene, which greatly improves the coupling level of human society. Social distancing and isolation have been widely introduced to counter the COVID-19 pandemic. It is demonstrated that a strategic social network-based reduction of contact strongly enhances the effectiveness of social distancing measures while keeping risks lower [15]. The risk of a public health crisis spreads all over the nerve endings of the world, connecting with each other, leading to the whole body. During this crisis, lack of awareness, knowledge and preparedness would put people and health care staff at risk. The dilemma is how to pass the knowledge of the current disease’s statistics and its prevention to the general population at a rate equivalent to or better than the spreading epidemic. At the same time, a huge amount of health-threatening misinformation is spreading at a faster rate than the disease itself [16]. Social media and networks have a significant impact on spreading fear and panic related to the COVID-19 outbreak with a potential negative influence on people’s mental health and psychological well-being [17]. In the new era, the public health crisis has folded the old characteristics and strengthened the attribute strength of the characteristics, which present as prominent systematization, cross-border and uncertainty [18].

### 2.2. Governance Mode for Public Health Crisis: “One Plan, Three Mechanisms”

China’s public health crisis management involves the construction of an emergency management system. Before the SARS outbreak in 2003, the main functions of the emergency management mechanism were scattered in multiple departments, often relying on non-established organizations to coordinate the responsibilities of departments. For example, the establishment of temporary emergency headquarters showed the characteristics of simplification and decentralization. After SARS in 2003, in order to solve the problem of the fragmentation of emergency management systems and functions, China began to build a public health emergency response mechanism. In 2003, emergency management work was started. The executive meeting of the State Council deliberated and passed the “Regulations on Emergency Response to Public Health Emergencies”. In 2004, the emergency management system was established, and the provincial overall emergency plans were issued. In 2006, the State Council issued “the national overall emergency plan for public emergencies”. In 2007, the legal system construction of emergency management was adopted. The emergency response law of the People’s Republic of China was promulgated and implemented on November 1 of that year. Thus, the emergency management system based on the framework of “One Plan, Three Mechanisms” has been gradually established in China. In “One plan, Three Mechanisms”, the “one plan” refers to the emergency plan, and the “three mechanisms” refer to the emergency management system, management mechanism and legal system, respectively. As of 2007, all provinces (autonomous regions and municipalities directly under the central government) have formulated their own overall plans. In addition, 97.9% of the cities (prefectures) and 92.8% of the counties (cities) have formulated overall plans. The emergency management system of “unified leadership, comprehensive coordination, classified management, hierarchical responsibility and territorial management” has been basically established. The basic mode of “One Plan, Three Mechanisms” in public health crisis management has solved the chaos and disorder in crisis emergency management. This governance mode has been tested in the severe snow disaster in southern China in 2008, Yushu earthquake in 2010 in Qinghai and especially the serious railway traffic accident on Yongwen railway in 2011. Although it also exposed some problems, such as the insensitivity of the pre-warning mechanism, “One plan, Three Mechanisms” has been gradually improved in crisis management and response.

Public health crisis is closely related to national security crisis, and national security crisis is higher than public health crisis in political rank. Although they are not equal concepts, a public security crisis can easily evolve into a national security crisis under the trigger of sudden major public events if not handled properly. Therefore, we should think and grasp public health crisis management under the framework of the overall national security concept, and consciously use the holistic thinking of the overall national security concept to look at the relationship between public and national security.

### 2.3. Dilemma of Public Health Crisis Governance

The occurrence of a public health crisis can be divided into natural causes and man-made causes, while most public crises are the process of interaction between human causes and natural causes, such as earthquakes, floods, fires, explosions, meteorological disasters, geological disasters, major traffic accidents, terrorist attacks and infectious disease disasters [19]. On the one hand, the governance of public health crisis should conform to the natural law of crisis, on the other hand, we should also explore the governance law and experience from human governance rationality. In the traditional governance framework, “no matter what kind of typical model is adopted, we always adhere to the” all-round “emergency management, and the government plays the main, and leading role in the state of crisis”. The government has long been regarded as the only subject of crisis governance, and the main body has been solidified. As a result, the vitality of crisis governance is insufficient, and the one-way operation of power from top to bottom affects the efficiency of governance. The second is the lack of crisis governance ability. Especially in the network era, the supervision mechanism, the government media management literacy, the division of local interests and social mobilization affect the crisis governance ability, and the general attribution also focuses on several aspects to explain the impact of the crisis governance ability on public health crisis governance. In addition, the governance dilemma of a public health crisis also concerns the lack of a communication mechanism between government and society and the lack of social participation, the information delay caused by the poor communication between the government and the media, the two-sided emotion of the government for social organizations (both want to use and worry about growing) and how the government and enterprises solve the cooperation obstruction of enterprises as rational economic people, and stimulate enterprises’ social responsibility. The lack of social participation is related to many factors, such as non-governmental organization (NGO), public safety awareness and public psychological behavior. The participation of social forces in public health crisis does not form a coordinated response system.

The general attribution of the dilemma of public health crisis governance emphasizes the joint participation of multiple subjects and the importance of communication, which dispels the “centrality” of the government role in the subject of public health crisis management, and places many stakeholders in crisis management, including media, community, public and enterprises, to present an equal situation. In the structure of equality, the ideal model is to manage the risk through the interaction and mutual assistance of multiple subjects, and to disperse the crisis risk with the government. However, the sudden public health crisis itself does not necessarily have a significant impact on the public’s risk perception. The public and other subjects are more likely to watch the crisis process, with suspicious eyes in the trust, and observe how the governance technology of public health crisis and the government level work together.

## 3. Transparency

China’s public health crisis governance model, namely “One plan, Three Mechanisms”, essentially follows the chain law of stimulation–response. From the internal structure of society, a basic picture of China’s modernization construction since the reform and opening up is a “mixed structure”. The “field separation” and “structural transformation” caused by the reform of the market economy orientation lack the germination of autonomy in the development of civil society. They often use the power of the market to release their own vitality. On the contrary, it is the integration of technological governance and a pressure political system. The independence of social pluralistic subjects is weak, and the dependence on power is not only reflected in their own development, but also in the characteristics of psychological dependence and expectation. In terms of administrative style, the prismatic administrative paradigm determined by many transitional colors of the social structure and economic situation dominates the current domestic normal governance and abnormal governance.

The chain law of stimulation–response means that there is no seamless transition from the occurrence of public health crisis to the response to governance. The crisis response model based on “One plan, Three Mechanisms” needs to be stimulated by public health crisis events. Generally speaking, the time difference between the degree of stimulation given by a major public health crisis and the stress response should be narrower, and the chain length of the excitation response should be shortened as far as possible, so that the occurrence and management of a public health crisis are almost synchronous, which should be the most rational state of public health crisis governance. However, the most important factor that determines the excitation response time distance is not natural, but human factors. Looking back at COVID-19 in China, the most fundamental reason for its rapid spread and development is that in the chain of stimulus–response to the public health crisis, the reaction was slow and lead to the failure of early warning. After the outbreak of COVID-19, officials from many provinces and municipalities openly expressed guilt and self-blame. The secretary of the provincial party committee mentioned the epidemic and revealed the shortcomings of local governance systems and governance capacity [20].

## 4. Governance

The emergency, derivative and uncertainty characteristics of a sudden major public health crisis mean that the faster the response, the higher the efficiency, and the subject of this response should be the government. Since a public health crisis from the trigger to the end forms a field in which the government is in first place, the government’s behavior and choices determine the direction of the public health crisis [4]. The advantages of the government are reflected in the unified authority, full cooperation, information possession and control, which are incomparable to other non-governmental subjects. In China’s political structure, first of all, the gathering and unification of authority mainly comes from the rapid response of the Chinese Communist Party, and the government is the output and converter of policies. Unified high authority means that the core structure of crisis governance can use the resources. Secondly, the government should have scientific, reasonable and efficient cooperation while gathering high authority, and the core index of judging government assistance is speed. After all, precise bureaucracy determines the lowest benchmark level of cooperation. The speed of the government’s response determines whether the cooperative weight of the government can get the opportunity in the chain of stimulation reaction. In addition, it is very important for the government to possess and control the information in the public health crisis field, which is not only reflected in the guidance of public opinion, but also reflected in open and transparent information sharing. After all, the public health crisis field is not a closed space, but open from beginning to end. The interaction and sharing of crisis information in and out of the field is very important. Any attempt to prevent communication between the field and the outside is futile. The result can only increase the risk level of the public health crisis in the theater domain, and the formation of information pressure outside the field will squeeze the space of the public health crisis field, and the governance flexibility of public health crisis will also be affected.

The key segments of the COVID-19 epidemic prevention and control process were reviewed and analyzed. In the response to the public health crisis, the warning failed, and the performance of local governments attracted huge attention. In public health crisis, although the advantages of the government are reflected in the gathering and unified authority, full cooperation and information control, the centralized authority of the government often occurs in the specific governance of public health crisis events, and the government’s cooperation and information control have already appeared in the pre-warning stage of the crisis. In China’s governance structure, the governance of public health crisis is a top-down decision-making mechanism. The information gathering and transmission from the bottom up should be selectively leaked. COVID-19 cases were discovered in the weeks before the outbreak. However, the bureaucracy at all levels has not played a role and the relevant departments have not been highly alert. The top-down decision-making mechanism in China’s governance structure is bound to match the concentration of power. Although the concentration of power has the advantage of concentrating on major events, it has a tense relationship with the effectiveness and autonomy of local governance. The flow of the government decision-making implementation mechanism is from top to bottom, and then extends to the horizontal level of government. In the major public health crisis, the vertical government hierarchy is combined. As the center of government cooperation, the contradiction and tension between the centralization of power and the autonomy and effectiveness of local governance have become the fundamental obstacle to the vertical-level cooperation of the government. The local government has the most comprehensive and truthful information of the new crown pneumonia, but it is far away from the level of decision-making and executive ability relative to the information it receives. The governance structure of “strong central weak place” has caused the information asymmetry and power and responsibility of the government hierarchy. COVID-19 is a major public health crisis, which requires the central government to cooperate efficiently and quickly. The time for cooperation between the central and local governments is very limited. The failure of an early warning of the COVID-19 epidemic crisis and the missed gold prevention and control period indicate that the collaboration between the government levels has been temporarily broken. For example, the official of the mayor of Wuhan claimed that without authorization, he could not disclose the epidemic situation to the public, which to some extent verified the tension between power concentration and effective local governance in China’s governance structure. In the face of a major public health crisis, the vertical cooperation at the government level is the theme, and the cooperation at the government level also involves the horizontal cooperation between local governments.

The response and decision-making optimization of a public health crisis involve many contents, such as the optimization of an epidemic prevention and control system, the strengthening of the emergency management ability, the professional background of local decision-makers, the blocked link between public health and medical institutions, serious brain drain in public health and insufficient medical supply and strategic reserve. Therefore, the response to public health crisis and the optimization of a decision-making mechanism can be said to be all inclusive, while from the perspective of government-level cooperation, China’s governance structure and government’s information possession and control are mainly considered, because the operation of the government level (especially the vertical relationship between central and local governments) in the governance structure of public health crisis is synchronous with the transmission and sharing of information. Once the information leakage occurs, it will affect the benign operation of the governance structure, and the efficient cooperation of different government levels and the government’s information possession, control and sharing will contribute to the response to public health crisis and the optimization of a decision-making mechanism. In addition, the crisis field formed after the occurrence of a public health crisis should pay more attention to the value of order. A chaotic crisis response order is an inefficient form of governance, which will eventually affect the resilience construction of the scene carrier of the crisis.

First of all, the tension between the centralization of power and effective local governance in China’s governance structure should be viewed rationally. On the one hand, we should not damage the superb overall control ability and pragmatic and efficient decision-making ability in the current governance pattern. On the other hand, we should build a flexible tension network between the central government and the local government to improve the vertical cooperation level of the government level. The asymmetry between the two sides of central and local vertical power needs to pay attention to the reasonable distribution and flow of power [12]. In major public health crisis events, the active release and downward movement of power can enable local governments to have sufficient emergency response capacity in the early stage of a public health crisis, and make a timely response by virtue of the advantages of neighboring crises, and the relative balance of vertical power distribution can also make both central and local governments. The relationship between power and responsibility in cooperation is further clarified and scientific. The tension between the centralization of power and effective local governance needs to be eased in the process. In particular, the experience accumulation of a major public health crisis and pilot areas is conducive to the construction of a tension network between the two, but the relationship between the central and local governments is not either one or the other. Therefore, while actively empowering, firstly, we should build a responsibility sharing mechanism to deal with the crisis, forming a governance network with unified decision-making, division of responsibilities and orderly operation; secondly, we should divide responsibilities according to the basic principle of the same rights and responsibilities, local party committees are responsible for organizing and leading, governments at all levels are responsible for coordination and cooperation and various social organizations should give full play to their advantages of connecting with the masses. The general public should also become active participants and contributors. As a normal resident, coronavirus whistleblower Doctor Li Wenliang was an active participant and contributor to the understanding and warning of COVID-19 in the early period. It can be seen that the function of thousands of the general public will be enlarged, and most importantly, this will make a contribution to the need of keeping a fast response to the public health crisis.

Secondly, the premise of the possession and control of government information is the disclosure and sharing of information [21,22]. The level of cooperation of the government should effectively open and share the relevant information of the public health crisis throughout the whole process, whether horizontally or vertically. The crisis field formed by the occurrence of a public health crisis is an open space. The local government, especially the local government nearest to the crisis event, will occupy and control the crisis information mechanically and selectively filter the information, which will seriously affect the interaction and cooperation with the central government. Due to the openness of the public health crisis field, crisis information spreads everywhere. When the public comes into contact with the overflowing information, especially when it is different from the local government’s information disclosure, the credibility of the local government will be widely doubted. The loss of trust between the government and the public will weaken the potential of cooperation that has existed or has been ready to be released. In the early warning stage before the outbreak of a public health crisis, the effective sharing of information is very important. It can not only promote the rapid cooperation between the horizontal and vertical levels of the government, but also warn the society, improve the public’s awareness of risk protection, mobilize social organizations and individuals, build a temporary “cloud ground” supervision mechanism for the implementation of public power online and offline, wake up the dormant power in the public health crisis response system and timely promote information transmission, consultation and decision-making. In addition, the effective sharing of information can also help the public to build psychological expectations in advance, calm the social crisis and panic, prevent irrational behaviors caused by blind emotions from damaging the overall situation of public health crisis management and repair the process of psychological trauma caused by the public health crisis.

The emergence of a public health crisis and the subsequent governance should focus on the core value of order. Many characteristics of public health crisis make the subject and governance process in the field prone to chaos, but the pursuit of order should be the value goal from the beginning to the end [23]. The flexibility, resilience and order of China’s governance structure are inseparable. In response to a public health crisis, the rapid construction of an order process, from the establishment of simple order elements to the initial construction of an order network to the final formation of an order structure, if the construction of order is general, timely build the governance order of public health crisis, and carry it out according to the changes in the public health crisis response environment. In the adjustment of an order structure, which also tests the hierarchical cooperation of the government and the control and sharing of information, the construction of an order governance structure and rapid control of a crisis should be placed in the high position of the crisis evaluation spectrum. On the one hand, from the perspective of the central government, the decisive control of the crisis situation and the rapid recovery of the order in the crisis areas have become the primary concern, which is naturally the first choice from the perspective of national governance. On the other hand, paying attention to the value of order is also the requirement of accommodative resilient governance. The “space field” of public health crisis contains the characteristics of tension, pressure, collusion, unipolar subject promotion and social appreciation. Therefore, the imagination to solve this dilemma needs to get rid of the highly tense “space field”, that is, to try to alleviate the tension of central and local governance structures and the asymmetry of information, rights and responsibilities that may appear in the trend of a traditional governance structure. Accommodative governance emphasizes that those (people or things) accepted in a fixed space and time range can be compatible with each other among different subjects and things. Compared with the highly tense “space field” of the traditional governance structure, accommodative governance is a more elastic and tension structure. With the help of accommodative governance, the “space field” of public health crisis governance can be divided, which can make public health crisis governance. At the same time, it pays more attention to the connotation of democracy and fairness [24].

## 5. Conclusions

Although the basic mode of public health crisis response in China, “One plan, Three Mechanisms”, has experienced many years of development and improvement, its essence is still a chain action of stimulation–response. There is an objective time difference between excitation and response itself, and the response to public health crisis is committed to shortening the chain distance between excitation and response, especially in the early warning stage of a public health crisis. Traced back to the outbreak of COVID-19, the tension between the power concentration and local effective governance in the development of the epidemic has temporarily broken the cooperation of the government, resulting in the failure of the early warning stage of the epidemic and then entering the outbreak stage directly. Under the high-pressure political system, the local government’s series of public health crisis response behaviors give us a study of the original causes of China’s governance structure. The concentration of power also brings high authority cohesion and the establishment of a high-level temporary command and leading group. The local government level replicates this behavior. For example, the central leading group of the new central coronavirus infection pneumonia outbreak was set up under the leadership of the Standing Committee of the Central Political Bureau, and the national epidemic prevention and control system was strengthened. One leadership and unified command are conducive to cross-system and all-round mobilization of resources and human resources, forming a huge energy to overwhelm the epidemic situation and conducive to the response to the public health crisis. China’s public health crisis governance structure is constantly adjusting, resulting from the development of a socialist market economy, the growth of civil society and the rise of social organizations. Further, major public health crisis events indirectly become an important force to promote the reform of China’s governance structure. We look forward to this major public health crisis to give us further answers.

## References

[1] Abrams E.M., Szefler S.J. (2020). COVID-19 and the impact of social determinants of health. Lancet Respir. Med..

[2] Ahmed F., Ahmed N., Pissarides C., Stiglitz J. (2020). Why inequality could spread COVID-19. Lancet Public Health.

[3] Anner M. Abandoned? The Impact of Covid-19 on Workers and Businesses at the Bottom of Global Garment Supply Chains. Center for Global Workers’ Rights Research Report (27 March 2020). https://www.workersrights.org/wp-content/uploads/2020/03/Abandoned-Penn-State-WRC-Report-March-27-2020.pdf.

[4] Blair R.A., Morse B., Tsai L.L. (2017). Public health and public trust: Survey evidence from the Ebola Virus Disease epidemic in Liberia. Soc. Sci. Med..

[5] Bollyky T.J., Templin T., Cohen M., Schoder D., Dieleman J.L., Wigley S. (2019). The relationships between democratic experience, adult health, and cause-specific mortality in 170 countries between 1980 and 2016: An observational analysis. Lancet.

[6] Chan J.F.-W., Yuan S., Zhang A.J., Poon V.K.-M., Chan C.C.-S., Lee A.C.-Y., Fan Z., Li C., Liang R., Cao J. (2020). Surgical mask partition reduces the risk of non-contact transmission in a golden Syrian hamster model for Coronavirus Disease 2019 (COVID-19). Clin. Infect. Dis..

[7] Chau T.H., Wan K.-M. (2020). Pour (Tear) Gas on Fire? Violent Confrontations and Anti-Government Backlash in Hong Kong. SSRN Electron. J..

[8] Cheng V.C.-C., Wong S.-C., Chuang V.W.-M., So S.Y.-C., Chen J.H.-K., Sridhar S., To K.K.-W., Chan J.F.-W., Hung I.F.-N., Ho P.-L. (2020). The role of community-wide wearing of face mask for control of coronavirus disease 2019 (COVID-19) epidemic due to SARS-CoV-2. J. Infect..

[9] Petrelli F., Grappasonni I., Peroni A., Kracmarova L., Scuri S. (2018). Survey about the potential effects of economic downturn on alcohol consumption, smoking and quality of life in a sample of Central Italy population. Acta Bio Med. Atenei Parm..

[10] Gianfredi V., Balzarini F., Gola M., Mangano S., Carpagnano L.F., Colucci M.E., Gentile L., Piscitelli A., Quattrone F., Scuri S. (2019). Leadership in Public Health: Opportunities for Young Generations Within Scientific Associations and the Experience of the “Academy of Young Leaders”. Front. Public Health.

[11] Cowling B.J., Ali S.T., Ng T.W.Y., Tsang T.K., Li J.C.M., Fong M.W., Liao Q., Kwan M.Y., Lee S.L., Chiu S.S. (2020). Impact assessment of non-pharmaceutical interventions against coronavirus disease 2019 and influenza in Hong Kong: An observational study. Lancet Public Health.

[12] Dionne K.Y. (2011). The Role of Executive Time Horizons in State Response to AIDS in Africa. Comp. Political Stud..

[13] Eimer T., Lütz S. (2010). Developmental states, civil society, and public health: Patent regulation for HIV/AIDS pharmaceuticals in India and Brazil. Regul. Gov..

[14] Gibson J.L., Caldeira G.A., Spence L.K. (2005). Why do people accept public policies they oppose? Testing legitimacy theory with a survey-based experiment. Political Res. Q..

[15] Block P., Hoffman M., Raabe I.J., Dowd J.B., Rahal C., Kashyap R., Mills M. (2020). Social network-based distancing strategies to flatten the COVID-19 curve in a post-lockdown world. Nat. Hum. Behav..

[16] Ahmad A.R., Murad H.R., Gardner M.R. (2020). The Impact of Social Media on Panic during the COVID-19 Pandemic in Iraqi Kurdistan: Online Questionnaire Study. J. Med. Internet Res..

[17] Heena S., Hunny S. (2020). Role of social media during the COVID-19 pandemic: Beneficial, destructive, or reconstructive?. Int. J. Acad. Med..

[18] Ho L.K.K. (2020). Legitimization & De-legitimization of Police. In British Colonial & Chinese SAR Hong Kong. J. Inter Reg. Stud. Reg. Glob. Perspect..

[19] Kraemer M.U.G., Yang C.-H., Gutierrez B., Wu C.-H., Klein B., Pigott D.M., Du Plessis L., Faria N.R., Li R., Hanage W.P. (2020). The effect of human mobility and control measures on the COVID-19 epidemic in China. Science.

[20] Tian H., Liu Y., Li Y., Wu C.-H., Chen B., Kraemer M.U.G., Li B., Cai J., Xu B., Yang Q. (2020). An investigation of transmission control measures during the first 50 days of the COVID-19 epidemic in China. Science.

[21] Wang C., Cheng Z., Yue X.-G., McAleer M. (2020). Risk Management of COVID-19 by Universities in China. J. Risk Financ. Manag..

[22] Wang C., Pan R., Wan X., Tan Y., Xu L., Ho C.S.H., Ho R.C.M. (2020). Immediate Psychological Responses and Associated Factors during the Initial Stage of the 2019 Coronavirus Disease (COVID-19) Epidemic among the General Population in China. Int. J. Environ. Res. Public Health.

[23] Zhang S., Wang Z., Chang R., Wang H., Xu C., Yu X., Tsamlag L., Dong Y., Wang H., Cai Y. (2020). COVID-19 containment: China provides important lessons for global response. Front. Med..

[24] Zhong B.-L., Luo W., Li H.-M., Zhang Q.-Q., Liu X.-G., Li W.-T., Li Y. (2020). Knowledge, attitudes, and practices towards COVID-19 among Chinese residents during the rapid rise period of the COVID-19 outbreak: A quick online cross-sectional survey. Int. J. Biol. Sci..

